# CRlncRNA: a manually curated database of cancer-related long non-coding RNAs with experimental proof of functions on clinicopathological and molecular features

**DOI:** 10.1186/s12920-018-0430-2

**Published:** 2018-12-31

**Authors:** Jun Wang, Xuan Zhang, Wen Chen, Jing Li, Changning Liu

**Affiliations:** 1 CAS Key Laboratory of Tropical Plant Resources and Sustainable Use, Xishuangbanna Tropical Botanical Garden, Chinese Academy of Sciences, Menglun, Yunnan 666303 People’s Republic of China; 2 Institute of Medical Sciences, Xiangya Hospital, Central South University, Changsha, 410008 People’s Republic of China; 3 University of Chinese Academy of Sciences, Beijing, 100049 People’s Republic of China

**Keywords:** Cancer, Long noncoding RNA, Database, Functional experiment, Clinicopathological feature

## Abstract

**Background:**

Recent studies demonstrated that long non-coding RNAs (lncRNAs) could be intricately implicated in cancer-related molecular networks, and related to cancer occurrence, development and prognosis. However, clinicopathological and molecular features for these cancer-related lncRNAs, which are very important in bridging lncRNA basic research with clinical research, fail to well settle to integration.

**Results:**

After manually reviewing more than 2500 published literature, we collected the cancer-related lncRNAs with the experimental proof of functions. By integrating from literature and public databases, we constructed CRlncRNA, a database of cancer-related lncRNAs. The current version of CRlncRNA embodied 355 entries of cancer-related lncRNAs, covering 1072 cancer-lncRNA associations regarding to 76 types of cancer, and 1238 interactions with different RNAs and proteins. We further annotated clinicopathological features of these lncRNAs, such as the clinical stages and the cancer hallmarks. We also provided tools for data browsing, searching and download, as well as online BLAST, genome browser and gene network visualization service.

**Conclusions:**

CRlncRNA is a manually curated database for retrieving clinicopathological and molecular features of cancer-related lncRNAs supported by highly reliable evidences. CRlncRNA aims to provide a bridge from lncRNA basic research to clinical research. The lncRNA dataset collected by CRlncRNA can be used as a golden standard dataset for the prospective experimental and *in-silico* studies of cancer-related lncRNAs. CRlncRNA is freely available for all users at http://crlnc.xtbg.ac.cn.

## Background

Cancer is a collection of diseases characterized by abnormal cell growth with the potential to invade adjacent tissues and spread to distant sites. Cancer formation is a complicated process that involves some common traits (cancer hallmarks), such as self-sufficiency in growth signals, insensitivity to anti-growth signals, evading apoptosis, limitless replicative potential, sustained angiogenesis, and tissue invasion and metastasis [1–3]. The discoveries of cancer driver protein-coding genes and their molecular mechanisms produced countless breakthroughs over the past years [4]. For example, many different types of cancer show a high incidence of TP53 mutations, leading to the expression of mutant p53 proteins [5]. While the genetic causes of cancer have been intensively studied, it is becoming clear that a large proportion of cancer susceptibility cannot be attributed to variation in protein-coding sequences [6]. On the other hand, a kind of length more than 200 nt non-coding RNA (long non-coding RNA, lncRNA) is found to be intricately implicated in cancer occurrence and development, and might work as effective therapeutic target and diagnostic marker for cancers with diverse types and phases [7–9].

Growing evidence reveals that lncRNAs play vital roles in a variety of biological processes such as cell differentiation [10, 11], apoptosis [12], autophagy [13], metabolism [14] and neoplasia [15], with the extremely diverse and complex mechanisms. LncRNAs can epigenetically modulate gene expression via recruiting chromatin modification factors [16–19]. In addition, lncRNAs can regulate mRNA translation and/or stability at post-transcription level by base-pairing with them [20, 21]. Besides, some lncRNAs can also function as miRNA sponges by titrating miRNAs away from their mRNA targets [22, 23]. Recent studies have shown that lncRNA disorders play an important role in the development and progression of cancer. For instance, ANRIL that has been reported to be dysregulated in several human cancers, is believed to facilitate the proliferation of cancer cells and repress apoptosis [24–26]. Another lncRNA, lncRNA-activated by TGF-beta (lncRNA-ATB), is an oncogene, which can promote the invasion-metastasis cascade in hepatocellular carcinoma [27]. LncRNA H19, an oncogene in diverse cancers, can promote cell proliferation by accelerating cell-cycle progression, and also function as miRNA sponge to antagonize the latter functions and lead to the de-repression of miRNA endogenous target [28–33].

Given a large number of cancer-related lncRNAs being discovered, some cancer-related lncRNA databases have been developed in recent years. For example, LncRNADisease [34] collects lncRNA-disease associations of both the experimentally reported and the computationally predicted. Lnc2Cancer provides more than 1000 manually curated associations between lncRNAs and human cancers [35]. TANRIC presents a resource of lncRNAs with clinical and other molecular data, both within and across tumor types [36]. Lnc2Catlas is an atlas of lncRNAs compiled with quantitative associations between lncRNAs and cancers using different computational methods [37]. LnCaNet serves as a comprehensive co-expression data resource of the interactions between lncRNA and non-neighboring cancer genes [38]. Although these databases are of immense help in the study of cancer-related lncRNAs, a database containing both clinicopathological and molecular features of cancer-related lncRNAs supported by highly reliable evidences, is of great importance to infuse lncRNA basic research into clinical research.

Here, we developed a new cancer-related lncRNA database, CRlncRNA, for two main objectives. Firstly, we anticipate providing a golden standard dataset for the follow-up experimental and *in-silico* studies of cancer-related lncRNAs. Other than the proceeding cancer-related lncRNA databases that collected most of data primarily from computational prediction or high-throughput experiments (e.g., differential expression data by sequencing or microarray), all entries in CRlncRNA were manually assembled from the published literature and supported by low-throughput functional experiments, which tend to provide more in-depth experimental evidence other than differential expression profiling. The second goal of this work is to mediate the transition from lncRNA basic research to clinical research. For each lncRNA in CRlncRNA, we gathered not only the information about its genomics location, epigenetic modification, expression profile and molecular interaction, but also the certificated clinicopathological features, such as correlative clinical stages and cancer hallmarks. Moreover, for better handy and effective use of CRlncRNA, we provided the tools for data browsing, searching and download, as well as the service of online BLAST, genome browser and gene network visualization. With this user-friendly interface as well as the highly valuable molecular and clinicopathological data, we believe CRlncRNA could serve as a productive tool for researchers in the field of lncRNA and cancer.

## Methods

### The data source for CRlncRNA

**Fig. 1:**
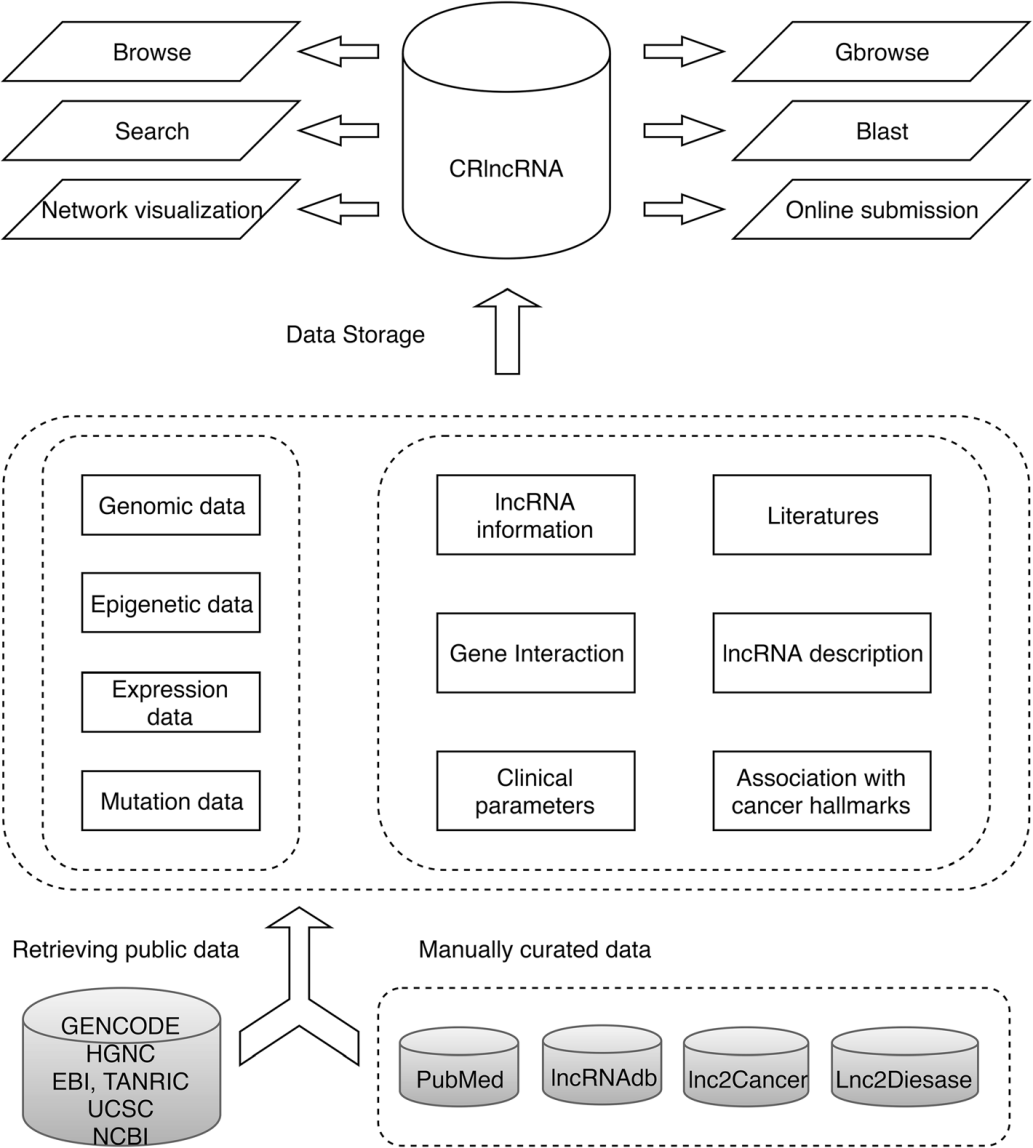
Overview of the architecture of CRlncRNA database. For each lncRNA entry, data were collected from three types of sources: the detailed information of annotation was manually assembled from published literature and cancer-related lncRNA databases; while others, such as genomic, epigenetic, expression and mutation information, came from other general databases. The lncRNAs in CRlncRNA can be accessed and analyzed by a variety of search and browse tools, as well as the services of online BLAST, genome browser and gene network visualization

An overview of CRlncRNA framework was shown in Fig. 1. First, we used ‘lncRNA’, ‘lincRNA’, ‘long noncoding RNA’ and ‘cancer’ as keywords to search PubMed database [39]. Then, the abstract and the full text of selected articles were manually screened to extract cancer-related lncRNAs and their detailed information of annotation, such as cancer type, binding factors, related cancer hallmarks. In addition, a summary description of each lncRNA was produced. In order to provide a comprehensive description, we also integrated information from lncRNAdb [40], lnc2Cancer [35] and lncRNADisease [34]. Only the lncRNAs that satisfy some specific criteria were adopted so as to ensure the high reliability of our dataset. That is, the selected lncRNAs were either differentially expressed in cancer (as verified by qRT-PCR), co-occurred with a significant pertinence to clinicopathological parameters (e.g., tumor differentiation, clinical stage, survival time); or else, were proven by functional experiments (e.g., colony formation assay, matrigel invasiveness assay, xenograft mouse model, and metastasis nude mouse model) to participate in cancer development.

To present more informative data, we first manually searched lncRNA IDs collected from the published literature through a selection of databases, such as HGNC, UCSC, NCBI, ENSEMBL, GENCODE, to get a corrected ID correspondence table. Next, we integrated genome-wide information from different general databases. For example, the histone modification data for H3K4me3 and H3K27ac markers were downloaded from ENCODE [41]. The phastCons conservation score, repeat elements and SNP information were downloaded from UCSC table browser [42]. LncRNA gene expression profiles were derived from Illumina human body map (https://www.ebi.ac.uk/gxa/experiments/E-MTAB-513/Results) and TANRIC database [36]. In addition, we manually collected the interactions between lncRNAs and proteins from literature, including direct and indirect, upstream and downstream interactions. In order to more effectively utilize CRlncRNA, we provided a variety of tools for data browsing, searching and download, as well as the services of online BLAST, genome browser and gene network visualization.

### Database architecture and implementation details

We developed a series of python scripts to import data in previous step to SQLite database step-by-step. In addition, we built a user-friendly web interface with Bootstrap (version 3.3.7) and jQuery (version 2.1.0) for users to query and visualize the collected data, as well as online servers. The principal functions of CRlncRNA include search, browse and network analysis. The network visualization was conducted by modifying the code from http://www.regulatorynetworks.org. The BLAST server was constructed by django-blastplus (version 0.4.0) and NCBI BLAST+ (version 2.3.0) [43]. We offered an interactive web-based platform for visualizing human genome datasets by use of Dalliance [44]. The web server of CRlncRNA runs on a dedicated Linux machine with the Nginx (version 1.9.9) and uWSGI (version 2.0.14). The server itself is a 4 Intel(R) Xeon(R) CPU E5–2640 v3 @ 2.60GHz with 8 Gigabytes of RAM. The application architecture consisted of several python web application according to the Django framework (version 1.9). CRlncRNA is supported by main standard-compliant web browsers such as Firefox, Google Chrome, Internet Explorer and Safari.

## Results

### Browsing and searching the database

**Fig. 2:**
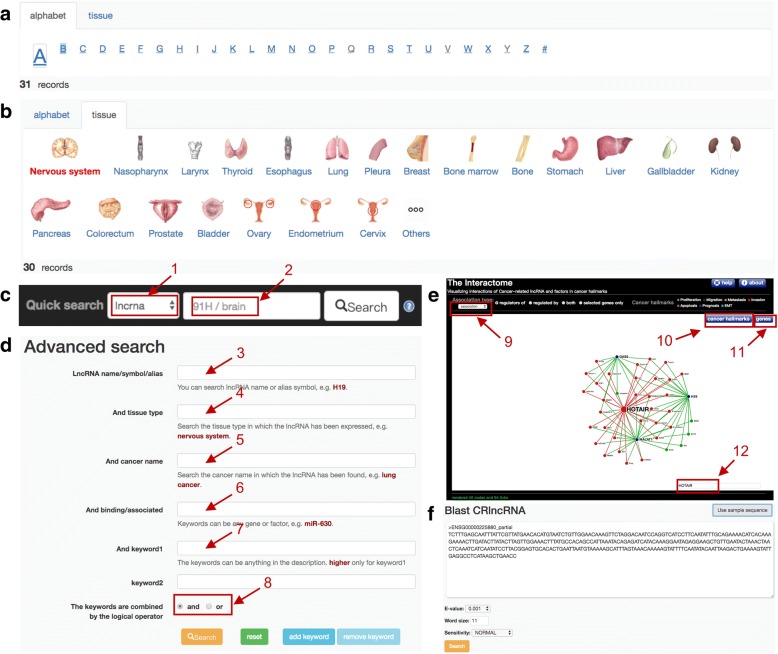
Screenshots of the browse and search pages. **a** Browsing lncRNA by alphabets. **b** Browsing lncRNA by tissues. **c** Quick searching. The drop-down list box enables users to search for one specific type of keyword (1, 2), such as lncRNA name, tissue type, cancer type etc. **d** Advanced searching. Different types of keywords can be combined to search (3–7), and the keywords can be combined with the logical operator ‘and’ or ‘or’ (8). **e** Network visualization service. Users can alternatively display specific sub-networks, by selecting specified types of interactions (9), cancer hallmarks (10) and one or more genes interested (11). Built-in network search function (12). **f** Web-based BLAST server

Users can browse all cancer-related lncRNAs directly on the ‘Browse’ page. There are two ways for browsing in CRlncRNA: (1) the way of ‘By alphabet’ will present lncRNAs in alphabetical order (Fig. 2a); while (2) ‘By tissue type’ will arrange lncRNAs according to their expressed tissues (Fig. 2b). Correspondingly, in the ‘Home’ page, there also provides a quick entry for browsing through the hyperlinks in ‘By alphabet’ and ‘By tissue type’.

CRlncRNA provide two kinds of keyword search services. One is the ‘Quick Search’ (on the top menu, Fig. 2c), in point to keywords including lncRNA name, tissue type or cancer type. The ‘Advanced Search’ (in the ‘Search’ page, Fig. 2d) performs a combined search. For example, you can find lncRNAs which were expressed in brain and have a relationship with glioma by inputting ‘brain’ in ‘tissue’ input box and ‘glioma’ in ‘cancer’ input box.

In addition, CRlncRNA also has web-based BLAST server for sequence similarity search (Fig. 2f), to assist users in identifying and annotating novel lncRNA transcripts.

### Network visualization service

Apart from the cancer-related lncRNAs, CRlncRNA also collected 1238 interactions between cancer-related lncRNAs and various factors (including RNAs and proteins), all of which are supported by experimental evidences. Users can visualize this complicated network using the network visualization service in ‘Network’ page (Fig. 2e). They can alternatively retrieve different types of interactions by the option of ‘Association type’ drop-down menu; for example, ‘all’ for all interactions, ‘binding’ for direct interactions, while, ‘association’ for indirect associations. The ‘cancer hallmarks’ button is used for selectively exhibiting the specific cancer-hallmark-related sub-network (the default value is proliferation sub-network). There are 7 cancer hallmarks to be chosen: proliferation, migration, metastasis, invasion, apoptosis, prognosis and EMT (epithelial-mesenchymal transition). The ‘genes’ button could assign one or more genes interested to display their sub-network. In addition, the built-in search function in the network visualization service enables the rapid position of a particular gene in the network.

### The detail information page

**Fig. 3:**
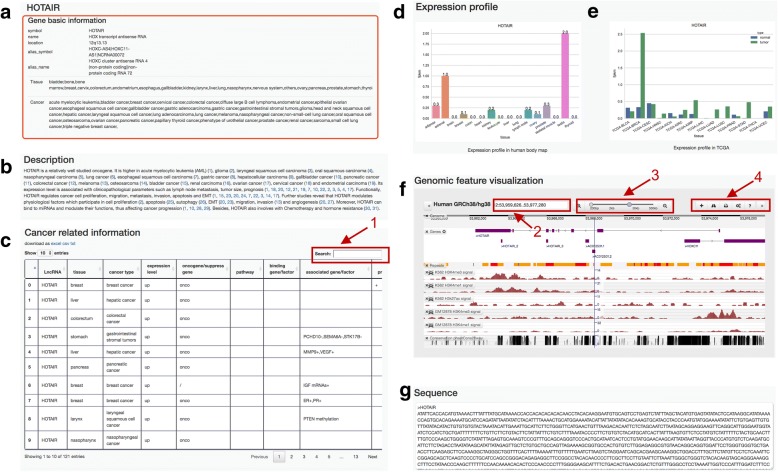
The detail information page. **a** The basic information of gene. **b** The description of lncRNA. **c** The cancer-related information, with the built-in keyword search function (1). **d** The lncRNA expression profile in normal tissues. **e** The lncRNA expression profile in cancer and paracancerous tissue pairs. **f** The genome browser, by which users can find one specific gene by using its genomic location or gene name (2), or quickly zoom to different levels of genomic resolution from a single base pair to a chromosome (3), or use the built-in toolbar to adjust the display (4). **g** The FASTA format sequence

**Fig. 4:**
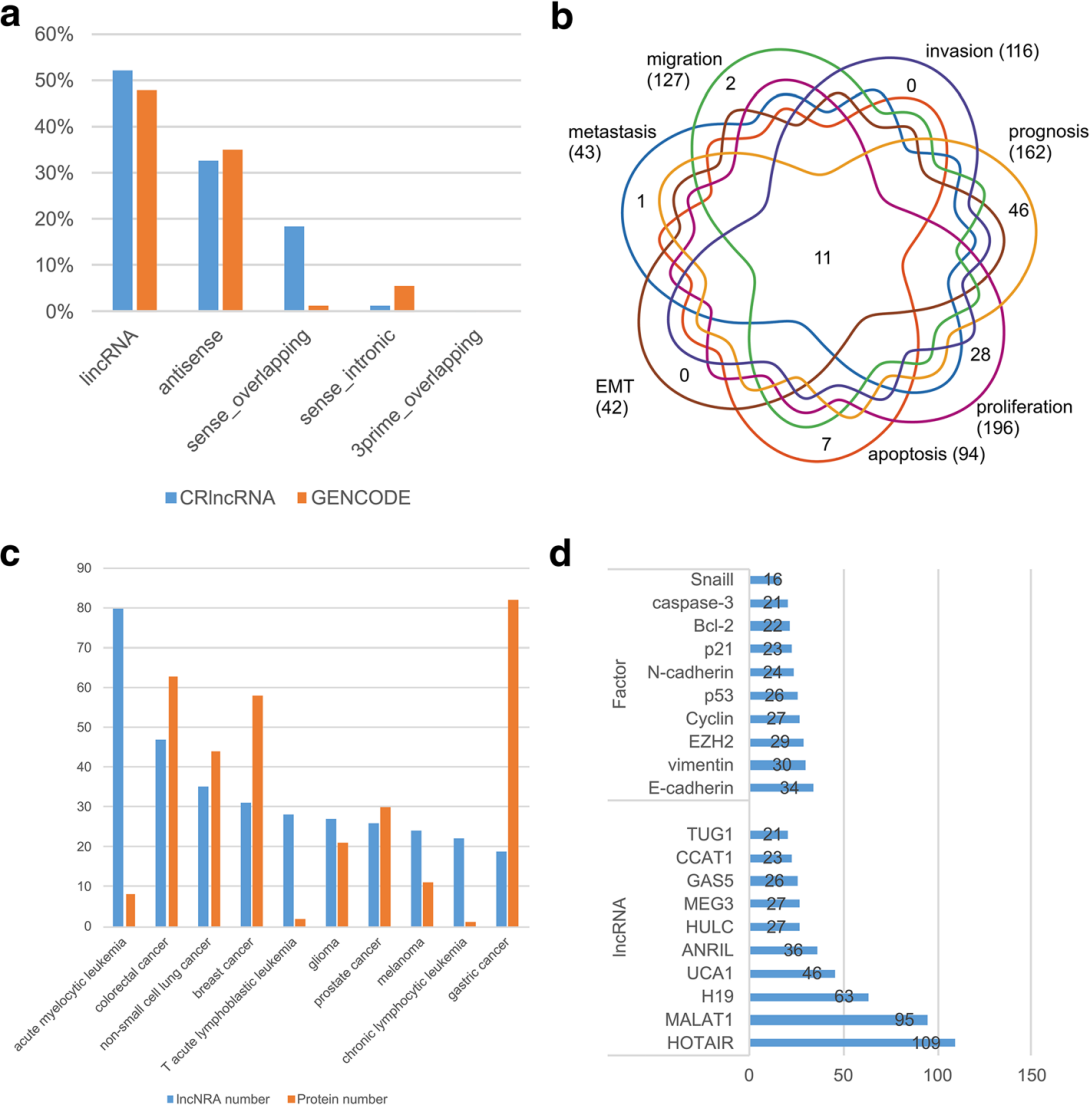
Statistics of the database content. **a** Distribution of different lncRNA subtypes in CRlncRNA (blue) and GENCODE (red) based on their genomic locations. **b** Venn diagram of the number of lncRNA related to different cancer hallmarks. **c** The number of reported tumor-related lncRNAs in the top ten enriched cancers (blue), referring to the number of the reported tumor-related proteins in the same cancer (red, data collected from Cancer Gene Census, https://cancer.sanger.ac.uk/census). **d** Top ten hub lncRNAs and hub factors in the cancer-related lncRNA network

For each lncRNA, CRlncRNA intended to collect the comprehensive information as much as possible. The detailed information page for a specific lncRNA can be accessed by clicking on the lncRNA name. As shown in Fig. 3, the collected information includes the following parts: (1) gene basic information, such as lncRNA symbol, location, the relevant tissues and cancer types (Fig. 3a); (2) the description of lncRNA, which contains a paragraph of detailed description on the lncRNA’s function and its clinicopathological and molecular features, as summarized from literature (Fig. 3b); (3) the cancer-related information, such as cancer type/pathway/hallmark, which is organized in tabular form and can be sorted and searched (Fig. 3c); (4) the lncRNA expression profiles in 16 normal tissues from Illumina human body map (Fig. 3d); (5) the lncRNA expression profiles in 14 cancer and paracancerous tissue pairs from TANRIC database (Fig. 3e); (6) the genome browser, which includes different annotation tracks (gene structure, epigenetics data, revolutionary conversion, etc.) (Fig. 3f); (7) the FASTA format sequence (Fig. 3g).

### Statistics of the database content

In the current version of CRlncRNA, we collected 355 entries of cancer-related lncRNAs, covering 1072 cancer-lncRNA associations with regard to 76 types of cancer, and 1238 interactions relevant to lncRNAs and different factors (RNAs and proteins). Compared with other cancer-related lncRNA databases (Table 1), CRlncRNA worked on providing a golden standard dataset for the later experimental and *in-silico* studies of cancer-related lncRNAs, thereby, adopted the most rigid standards for data acquisition. Moreover, CRlncRNA provided more full-scale annotations compared with others, particularly in the aspect of clinicopathological information, which is valuable for better translation from the basic research of cancer-related lncRNA to clinical research.

**Table 1:** Comparison of CRlncRNA with other cancer-related lncRNA databases

Database	lncRNA number	Basic data requirement	lncRNA sequence	Expression profile	Gene interaction	Cancer hallmarks	Clinical parameters
Lnc2Cancer	666	Expression^a^	×	×	×	×	×
lnCaNet	9641	Expression^b^	√	√	√	×	×
lncRNADisease	2478	Prediction^c^	√	×	√	×	×
Lnc2Catlas	27,670	None^d^	√	√	√	×	×
TANRIC	12,727	None^e^	×	√	√	×	√
CRlncRNA	355	Functional^f^	√	√	√	√	√

^a^Differential expression between cancer and normal samples

^b^Co-expression with cancer-related protein-coding genes

^c^More than half of the collected lncRNAs are predicted base on its genomic context

^d^All lncRNAs from GENCODE database

^e^All intergenic lncRNAs from GENCODE database

^f^Low-throughput functional experiments

### Distribution of different lncRNA subtypes

According to the gene/transcript biotype classification system in GENCODE & Ensembl (https://www.gencodegenes.org/pages/biotypes.html), we counted the distribution of different lncRNA subtypes based on their genomic location (Fig. 4a). Similar to those collected in GENCODE, the vast majority of cancer-related lncRNAs could be classified as ‘lincRNA’ and ‘antisense’, while the lncRNAs of ‘3prime_overlapping’ are less than 1%. On the other hand, the percentage of ‘sense_overlapping’ cancer-related lncRNA is obviously higher than in GENCODE (18.3% vs. 1.19%), while the percentage of ‘sense_intronic’ cancer-related lncRNA is obviously lower than in GENCODE (1.37% vs. 5.77%).

### LncRNAs related to different cancer hallmarks

We added up the number of cancer-related lncRNA associated with different cancer hallmarks (Fig. 4b). In total, there are 116, 127, 43, 42, 94, 196 and 162 lncRNAs related to ‘invasion’, ‘migration’, ‘metastasis’, ‘EMT’, ‘apoptosis’, ‘proliferation’ and ‘prognosis’, respectively. The most noteworthy is there are 11 lncRNAs (SPRY4-IT1, GAS5, LINC01133, HOTAIR, TUG1, ROR, MALAT1, H19, BANCR, NEAT1, HOTTIP) associated with all of the seven cancer hallmarks. In addition, the Venn diagram demonstrated that, despite the fact that there is an obvious overlapping between different hallmarks, a lot of unique lncRNAs emerged in hallmarks ‘prognosis’ (46 lncRNAs) and ‘proliferation’ (28 lncRNAs).

### Cancer-related lncRNAs in the top ten enriched cancers

We also counted the number of the reported cancer-related lncRNAs in the top ten enriched cancers (Fig. 4c). Compared with cancer-related lncRNAs, the studies on cancer-related proteins were far more comprehensive. Correspondingly, for some cancer types, such as gastric cancer, breast cancer, and colorectal cancer, it is not unexpected that the number of the reported cancer-related proteins is greater than that of lncRNAs in the same cancer. But in the cancer types of acute myelocytic leukemia, T acute lymphoblastic leukemia, chronic lymphocytic leukemia, glioma and melanoma, we were surprised to see that the amount of the known cancer-related lncRNAs had much exceeded that of proteins, especially in three types of leukemia. For example, 80 lncRNAs are associated with acute myelocytic leukemia, while only 8 proteins relevant to it.

### Hub lncRNAs in the cancer-related lncRNA network

It is apparent that there was a complicated interaction network between cancer-related lncRNAs and various factors (RNAs and proteins). Hence, we systematically summarized the top ten hub lncRNAs and hub factors. As shown in Fig. 4d, the list of top ten hub factors contained many vital proteins (e.g., cadherin, vimentin and EZH2) associated with cellular signaling, cell migration and invasion, and epigenetic regulation, and other star genes in the cancer studies (like p53, p21). While in the list of top ten hub lncRNAs, HOTAIR, MALAT1 and H19 are ranking the first three, wherein HOTAIR is the biggest hub node in the network and linked with 109 factors (including P53, P21 and P16). HOTAIR is a relatively well-studied oncogene, its expression level in cancer is an efficient predictor of metastasis and survival [9, 45, 46].

## Discussion

More and more studies on cancer-related lncRNAs have identified the crucial roles of lncRNAs in cancer processes [4, 47]. Thereby, it is very important to summarize and integrate the information of these cancer-related lncRNAs for conducting the subsequent clinical studies. However, it is worth to think how to achieve this goal. Here, we confined the cancer types strictly within those malignant tumors with the exclusion of benign tumors that are usually localized and do not spread to other parts of the body. For example, pituitary adenoma and neurofibromatosis type 1, which had been included in Lnc2Cancer database [35], are non-cancerous and benign tumors. The second question is how to define cancer-related lncRNA. Due to the excessive false positive, the data of cancer-related lncRNAs, if only with evidences from prediction or high-throughput experiments, are likely insufficient for the researches afterward; particularly when utilizing these data for developing machine learning approaches, it’s not instrumental. Considering that, we persist in two criteria for constructing CRlncRNA: (1) Only targeting cancer -- the malignant tumor; (2) Strict inclusion standard, only including lncRNAs that have low-throughput functional experiments evidence. We hope that our CRlncRNA database could provide a golden standard dataset for future cancer-related lncRNA studies.

In CRlncRNA, we collected many molecular and clinicopathological data about cancer-related lncRNAs, which may provide references and insights for investigating lncRNA’s roles in cancer. During the preliminary statistical analysis, we found that the percentage of ‘sense_overlapping’ cancer-related lncRNAs is remarkably above the average of all lncRNAs (nearly more than 15 times), which would potentially facilitate the studies of cancer-related lncRNAs on the identification of molecular mechanisms and the development of novel prediction algorithm. In addition, there are 11 lncRNAs related to all the seven cancer hallmarks when we tested the relevance between cancer-related lncRNAs and cancer hallmarks. These lncRNAs may play important roles in cancer occurrence and development, and may be the most valuable targets for cancer therapy. A much more interesting result came from the quantitative comparison between reported cancer-related lncRNAs and proteins in different cancer types. Despite just the beginning of studies of cancer-related lncRNAs, the amount of lncRNAs in some cancer types (like acute myelocytic leukemia, T acute lymphoblastic leukemia, chronic lymphocytic leukemia) is much more than that of proteins, implying lncRNAs can be potentially as novel biomarkers for blood cancer diagnosis and therapy.

In order to better promote the cancer-related lncRNA studies, CRlncRNA provided a variety of web-based tools. For example, gene network visualization service can help to survey lncRNA-involved interaction network, genome browser to search and visualize for lncRNA’s genomic neighborhood. In the future, we will add more online tools in CRlncRNA. For example, we are exploiting the CRlncRNA data to develop an algorithm for predicting cancer-related lncRNAs, which will provide the online service before long. In addition, other than periodically retrieving the freshly-published literature, we plan to integrate more clinicopathological information in CRlncRNA in the aspect of data collection, such as survival, radiation sensitivity and drug resistance. The study of cancer-related lncRNA had been a multi-discipline crossed hot field, which would be bound to affect our conception of cancer, from its causative origins to the design and prescription of treatments, profoundly. Meanwhile, this field is still in its infancy, and we are far from comprehending lncRNAs and incorporating it into the clinical application. We believe that a rigid standard for cancer-related lncRNA database, with multiple data sources and more online services, will offer an important platform for further study and more pronounced understanding of lncRNA functions and mechanisms both in physiological and pathological condition.

## Conclusions

In this work, we presented CRlncRNA, a manually curated database for the cancer-related lncRNAs, which offers not only the experimental validated molecular mechanisms and the related clinicopathological features of collected items, but also many useful tools to perform data-mining. The aim of CRlncRNA is to provide a more orientated curated database that only targets to cancer -- the malignant tumor. In addition, for the sake of the identification of cancer-related lncRNAs in future, CRlncRNA could provide golden standard datasets which are highly credible and well-informed for the development of tools and methodology.

## Abbreviations

EMT: Epithelial–mesenchymal transition
lincRNA: Long intergenic non-coding RNA
lncRNA: Long non-coding RNA

## References

[1] Hanahan D, Weinberg RA (2000). The hallmarks of cancer. Cell.

[2] Hanahan D, Weinberg RA (2011). Hallmarks of cancer: the next generation. Cell.

[3] Fouad YA, Aanei C (2017). Revisiting the hallmarks of cancer. Am J Cancer Res.

[4] Schmitt AM, Chang HY (2016). Long noncoding RNAs in Cancer pathways. Cancer Cell.

[5] Muller PA, Vousden KH (2014). Mutant p53 in cancer: new functions and therapeutic opportunities. Cancer Cell.

[6] Cheetham SW, Gruhl F, Mattick JS, Dinger ME (2013). Long noncoding RNAs and the genetics of cancer. Br J Cancer.

[7] Cabili MN, Trapnell C, Goff L, Koziol M, Tazon-Vega B, Regev A, Rinn JL (2011). Integrative annotation of human large intergenic noncoding RNAs reveals global properties and specific subclasses. Genes Dev.

[8] Akers JC, Gonda D, Kim R, Carter BS, Chen CC (2013). Biogenesis of extracellular vesicles (EV): exosomes, microvesicles, retrovirus-like vesicles, and apoptotic bodies. J Neuro-Oncol.

[9] Bolha L, Ravnik-Glavac M, Glavac D (2017). Long noncoding RNAs as biomarkers in Cancer. Dis Markers.

[10] Mathieu EL, Belhocine M, Dao LT, Puthier D, Spicuglia S (2014). Functions of lncRNA in development and diseases. Med Sci (Paris).

[11] Fatica A, Bozzoni I (2014). Long non-coding RNAs: new players in cell differentiation and development. Nat Rev Genet.

[12] Rossi MN, Antonangeli F (2014). LncRNAs: new players in apoptosis control. Int J Cell Biol.

[13] Xiong H, Ni Z, He J, Jiang S, Li X, He J, Gong W, Zheng L, Chen S, Li B (2017). LncRNA HULC triggers autophagy via stabilizing Sirt1 and attenuates the chemosensitivity of HCC cells. Oncogene.

[14] Zhao XY, Lin JD (2015). Long noncoding RNAs: a new regulatory code in metabolic control. Trends Biochem Sci.

[15] Huarte M (2015). The emerging role of lncRNAs in cancer. Nat Med.

[16] Bergmann JH, Spector DL (2014). Long non-coding RNAs: modulators of nuclear structure and function. Curr Opin Cell Biol.

[17] Spitale RC, Tsai MC, Chang HY (2011). RNA templating the epigenome: long noncoding RNAs as molecular scaffolds. Epigenetics-Us.

[18] Davidovich C, Cech TR (2015). The recruitment of chromatin modifiers by long noncoding RNAs: lessons from PRC2. RNA.

[19] Zhao J, Sun BK, Erwin JA, Song JJ, Lee JT (2008). Polycomb proteins targeted by a short repeat RNA to the mouse X chromosome. Science.

[20] Gong C, Maquat LE (2011). lncRNAs transactivate STAU1-mediated mRNA decay by duplexing with 3’ UTRs via Alu elements. Nature.

[21] Abdelmohsen K, Panda AC, Kang MJ, Guo R, Kim J, Grammatikakis I, Yoon JH, Dudekula DB, Noh JH, Yang X (2014). 7SL RNA represses p53 translation by competing with HuR. Nucleic Acids Res.

[22] Salmena L, Poliseno L, Tay Y, Kats L, Pandolfi PP (2011). A ceRNA hypothesis: the Rosetta stone of a hidden RNA language?. Cell.

[23] Tay Y, Rinn J, Pandolfi PP (2014). The multilayered complexity of ceRNA crosstalk and competition. Nature.

[24] Zhang EB, Kong R, Yin DD, You LH, Sun M, Han L, Xu TP, Xia R, Yang JS, De W (2014). Long noncoding RNA ANRIL indicates a poor prognosis of gastric cancer and promotes tumor growth by epigenetically silencing of miR-99a/miR-449a. Oncotarget.

[25] Zhao JJ, Hao S, Wang LL, Hu CY, Zhang S, Guo LJ, Zhang G, Gao B, Jiang Y, Tian WG (2016). Long non-coding RNA ANRIL promotes the invasion and metastasis of thyroid cancer cells through TGF-beta/Smad signaling pathway. Oncotarget.

[26] Zhu HX, Li XC, Song YR, Zhang P, Xiao YJ, Xing YF (2015). Long non-coding RNA ANRIL is up-regulated in bladder cancer and regulates bladder cancer cell proliferation and apoptosis through the intrinsic pathway. Biochem Bioph Res Co.

[27] Yuan JH, Yang F, Wang F, Ma JZ, Guo YJ, Tao QF, Liu F, Pan W, Wang TT, Zhou CC (2014). A long noncoding RNA activated by TGF-beta promotes the invasion-metastasis cascade in hepatocellular carcinoma. Cancer Cell.

[28] Tsang WP, Ng EK, Ng SS, Jin H, Yu J, Sung JJ, Kwok TT (2010). Oncofetal H19-derived miR-675 regulates tumor suppressor RB in human colorectal cancer. Carcinogenesis.

[29] Luo M, Li Z, Wang W, Zeng Y, Liu Z, Qiu J (2013). Long non-coding RNA H19 increases bladder cancer metastasis by associating with EZH2 and inhibiting E-cadherin expression. Cancer Lett.

[30] Vennin C, Spruyt N, Dahmani F, Julien S, Bertucci F, Finetti P, Chassat T, Bourette RP, Le Bourhis X, Adriaenssens E (2015). H19 non coding RNA-derived miR-675 enhances tumorigenesis and metastasis of breast cancer cells by downregulating c-Cbl and Cbl-b. Oncotarget.

[31] Han D, Gao X, Wang M, Qiao Y, Xu Y, Yang J, Dong N, He J, Sun Q, Lv G (2016). Long noncoding RNA H19 indicates a poor prognosis of colorectal cancer and promotes tumor growth by recruiting and binding to eIF4A3. Oncotarget.

[32] Liu C, Chen Z, Fang J, Xu A, Zhang W, Wang Z (2016). H19-derived miR-675 contributes to bladder cancer cell proliferation by regulating p53 activation. Tumour Biol.

[33] Liang WC, Fu WM, Wong CW, Wang Y, Wang WM, Hu GX, Zhang L, Xiao LJ, Wan DCC, Zhang JF (2015). The lncRNA H19 promotes epithelial to mesenchymal transition by functioning as miRNA sponges in colorectal cancer. Oncotarget.

[34] Chen G, Wang Z, Wang D, Qiu C, Liu M, Chen X, Zhang Q, Yan G, Cui Q (2013). LncRNADisease: a database for long-non-coding RNA-associated diseases. Nucleic Acids Res.

[35] Ning S, Zhang J, Wang P, Zhi H, Wang J, Liu Y, Gao Y, Guo M, Yue M, Wang L (2016). Lnc2Cancer: a manually curated database of experimentally supported lncRNAs associated with various human cancers. Nucleic Acids Res.

[36] Li J, Han L, Roebuck P, Diao L, Liu L, Yuan Y, Weinstein JN, Liang H (2015). TANRIC: An interactive open platform to explore the function of lncRNAs in Cancer. Cancer Res.

[37] Ren C, An G, Zhao C, Ouyang Z, Bo X, Shu W (2018). Lnc2Catlas: an atlas of long noncoding RNAs associated with risk of cancers. Sci Rep.

[38] Liu Y, Zhao M (2016). lnCaNet: pan-cancer co-expression network for human lncRNA and cancer genes. Bioinformatics.

[39] Coordinators NR (2016). Database resources of the National Center for biotechnology information. Nucleic Acids Res.

[40] Quek XC, Thomson DW, Maag JL, Bartonicek N, Signal B, Clark MB, Gloss BS, Dinger ME (2015). lncRNAdb v2.0: expanding the reference database for functional long noncoding RNAs. Nucleic Acids Res.

[41] Consortium EP (2012). An integrated encyclopedia of DNA elements in the human genome. Nature.

[42] Speir ML, Zweig AS, Rosenbloom KR, Raney BJ, Paten B, Nejad P, Lee BT, Learned K, Karolchik D, Hinrichs AS (2016). The UCSC genome browser database: 2016 update. Nucleic Acids Res.

[43] Camacho C, Coulouris G, Avagyan V, Ma N, Papadopoulos J, Bealer K, Madden TL (2009). BLAST+: architecture and applications. BMC Bioinformatics.

[44] Down TA, Piipari M, Hubbard TJ (2011). Dalliance: interactive genome viewing on the web. Bioinformatics.

[45] Hajjari M, Salavaty A (2015). HOTAIR: an oncogenic long non-coding RNA in different cancers. Cancer Biol Med.

[46] Gupta RA, Shah N, Wang KC, Kim J, Horlings HM, Wong DJ, Tsai MC, Hung T, Argani P, Rinn JL (2010). Long non-coding RNA HOTAIR reprograms chromatin state to promote cancer metastasis. Nature.

[47] Prensner JR, Chinnaiyan AM (2011). The emergence of lncRNAs in cancer biology. Cancer Discov.

